# An Overview of the Posttranslational Modifications and Related Molecular Mechanisms in Diabetic Nephropathy

**DOI:** 10.3389/fcell.2021.630401

**Published:** 2021-05-28

**Authors:** Yu Cao, Zhao Yang, Ying Chen, Shuai Jiang, Zhen Wu, Baoping Ding, Yang Yang, Zhenxiao Jin, Haifeng Tang

**Affiliations:** ^1^Department of Chinese Materia Medica and Natural Medicines, School of Pharmacy, The Air Force Medical University, Xi’an, China; ^2^Department of Gynaecology and Obstetrics, The First Affiliated Hospital of Xi’an Jiaotong University, Xi’an, China; ^3^Department of Hematology, The First Affiliated Hospital of Xi’an Jiaotong University, Xi’an, China; ^4^Key Laboratory of Resource Biology and Biotechnology in Western China, Ministry of Education, Faculty of Life Sciences, Northwest University, Xi’an, China; ^5^Department of Cardiovascular Surgery, Xijing Hospital, The Air Force Medical University, Xi’an, China

**Keywords:** diabetic nephropathy, posttranslational modifications, pathogenesis, pathophysiology, therapeutic targets

## Abstract

Diabetic nephropathy (DN), a common diabetic microvascular complication, is characterized by its complex pathogenesis, higher risk of mortality, and the lack of effective diagnosis and treatment methods. Many studies focus on the diagnosis and treatment of diabetes mellitus (DM) and have reported that the pathophysiology of DN is very complex, involving many molecules and abnormal cellular activities. Given the respective pivotal roles of NF-κB, Nrf2, and TGF-β in inflammation, oxidative stress, and fibrosis during DN, we first review the effect of posttranslational modifications on these vital molecules in DN. Then, we describe the relationship between these molecules and related abnormal cellular activities in DN. Finally, we discuss some potential directions for DN treatment and diagnosis. The information reviewed here may be significant in the design of further studies to identify valuable therapeutic targets for DN.

## Introduction

Diabetic nephropathy (DN), one of the common microvascular complications of diabetes, is characterized by albuminuria (>300 mg/day) and a reduced glomerular filtration rate (GFR) (Pugliese, 2014). There has been a parallel increase in the incidence of diabetes and the health-related and economic consequences of DN since DN induces or aggravates other diabetic-related complications^1^ (de Boer et al., 2011; Valencia and Florez, 2017). Approximately 30–40% of patients with type 1 diabetes mellitus (T1DM) and 20–30% of patients with type 2 diabetes mellitus (T2DM) develop DN after a disease duration of 15–30 years (Fioretto et al., 2006; Fukuda et al., 2006). Furthermore, DN is a leading cause of the end-stage renal disease (ESRD) in developed countries and consequently diabetes-associated chronic kidney disease (CKD) (de Boer et al., 2011; Thomas et al., 2016). It is the major contributor to renal replacement therapy in Americans (de Boer et al., 2011). Decades of research have clarified that DN is characterized by the thickening of the glomerular basement membrane (GBM), podocyte effacement, tubulointerstitial fibrosis, and nodular extracellular matrix (ECM) accumulations (Kimmelstiel–Wilson lesions) in the glomerulus (Tzeng et al., 2013). Although recent advances emphasized the therapeutic value of sodium-dependent glucose transporters 2 (SGLT2) (Zelniker et al., 2019), inhibitors, and glucagon-like peptide 1 (GLP-1) (Kristensen et al., 2019) receptor agonist for DN and developing novel anti-diabetic agents that have facilitated optimum glycemic control, DN has not yet been cured, indicating the need for cause-specific treatments. The current therapies that relieve the severity of DN all focus on the abovementioned pathophysiological changes and include the treatment of hyperglycemia, hypertension, and dyslipidemia or the additional use of inhibitors of the renin–angiotensin system if the patient has significant albuminuria (Heerspink and de Zeeuw, 2011). Thus, the mechanisms underlying their renoprotective effects remain to be elucidated. This predicament is closely related to the complex pathogenesis of DN in multiple molecular regulation-related and abnormal cellular activities.

Posttranslational modifications (PTMs), one of the routes to proteome expansion, are covalent modifications that occur after DNA has been transcribed into RNA and translated into proteins. It is well known that the nascent or folded proteins are stable under physiological conditions; the process of performing a series of specific enzyme-catalyzed modifications on its side chain or backbone is known as PTM of a protein (Walsh et al., 2005). PTMs are highly dynamic and largely reversible and play a significant role in numerous cellular activities. Recent decades have seen great research progress on PTMs ranging from conventional PTMs such as phosphorylation and polyubiquitylation to unconventional PTMs such as methylation and acetylation. PTMs play a particular role in the occurrence and process of DN, which is closely related to the fact that PTMs relay rapid messages among cells via rapidly and reversibly modifying essential proteins (Anil Kumar et al., 2014; Barutta et al., 2015; Donate-Correa et al., 2015; Garten et al., 2015).

Nuclear factor kappa-B (NF-κB), nuclear factor erythroid-2 related factor 2 (Nrf2), and transforming growth factor-β (TGF-β), the core molecules in DN, are regulated to a great extent by PTMs (Table 1). However, the emerging role of PTMs in NF-κB, Nrf2, and TGF-β during DN has not explicitly been summarized, and the present review aims to fill this gap in the literature. In this review, we first summarize the effect of the most common PTMs on key molecules closely related to DN, including NF-κB, Nrf2, and TGF-β. Then, we describe the relationship between these molecules and related abnormal cellular activities, including inflammation, oxidative stress, and fibrosis in DN. Finally, the potential directions for DN treatment are discussed. This review presents a comprehensive picture of PTMs in DN and provides a valuable focus on the impending need for further early diagnostic and effective therapeutic studies of DN.

**TABLE 1:** The roles of central signaling pathways in the common abnormal cellular activities in DN.

Pathology	Targets	Central signaling pathways	
Inflammation	NF-κB	• IL-1β, IL-6, and TNF-α expression increased	• Elmarakby and Sullivan (2012), Sun and Kanwar (2015), Sun et al. (2016), and Yang et al. (2016)
		• Genes encoding TGF-β1, AKR1B1, MCP-1 (CCL2), ICAM-1 increased	• Park et al. (2000), Goldberg et al. (2006), Nam et al. (2008), and Yang et al. (2008)
		• PKCβ signaling, the RAS pathway, AGE accumulation and oxidative stress increased	• Kumar et al. (2001), Lee et al. (2004, 2010), Pillarisetti and Saxena (2004), Ohga et al. (2007), Zhang et al. (2007), Liang et al. (2010), Soetikno et al. (2011), and Li et al. (2018)
	MAPK	• Genes encoding pro-inflammatory molecules including TNF-α, IL-1β, and IL-6 increased	• Ma et al. (2009) and Tesch (2017)
Oxidative stress	Nrf2	• Generation of AGEs promoted	• Mangalmurti et al. (2010), Delbin et al. (2012), Lo et al. (2015), and Barandalla et al. (2017)
		• The renin–angiotensin system and expression of TGF-β, NF-κB, AP-1, and SP-1 dysregulated	• Shao et al. (2013)
Fibrosis	TGF-β	• EMT fibrosis promoted via numerous intracellular signals such as protein kinase and cytokines.	• Sutariya et al. (2016)
		• Phosphorylation of SMAD1/5 or MAD2/3 increased	• Massague (2000), Lebrin et al. (2005), Scherner et al. (2007), and Velasco et al. (2008)

## The Abnormal Cellular Activities and Core Molecules in DN

Increasing numbers of studies have strengthened the theory that the inflammatory response of DN is associated with abnormalities of renal glomerular and tubular epithelial, endothelial, and interstitial cells (Anil Kumar et al., 2014; Barutta et al., 2015; Donate-Correa et al., 2015; Garten et al., 2015). NF-κB, a vital factor that regulates the inflammatory response, serves as an agent in the complex inflammatory response system and other abnormal cellular activities. NF-κB participates in DN via its regulation by numerous upstream signals such as Silent mating type information regulation 2 homolog-1 (SIRT1) (Busch et al., 2012). Scholars reported that SIRT1 directly inhibits NF-κB via deacetylating the p65 subunit of the complex, while indirectly inhibiting NF-κB signaling by stimulating oxidative energy production via the activation of AMP-activated protein kinase (AMPK), peroxisome proliferator-activated receptor-α (PPAR-α), and PPAR gamma coactivator 1α (PCG-1α), finally suppressing inflammation (Rubattu et al., 2015; Lin et al., 2017). This relationship is related to the consequent downregulation of tumor necrosis factor-α (TNF-α) and TGF-β, which improved inflammation and fibrosis in DN (Lin et al., 2017). Additionally, the modulation of NF-κB is involved in abnormal oxidative conditions. NF-κB is a redox-sensitive transcription factor, of which the upregulation drove a pro-inflammatory shift that feedbacked oxidative stress (Kauppinen et al., 2013).

A persistent hyperglycemic state and an increase in advanced glycation end-products (AGEs) serve as the prime contributors to oxidative stress and the consequent overproduction of reactive oxygen species (ROS) (Miranda-Diaz et al., 2016). This oxidative condition, in turn, accelerates the generation of AGEs and the reaction between AGEs and the receptor for AGEs (RAGE), which strengthens oxidative stress (Coughlan et al., 2009). The complex interaction between oxidative stress and AGEs affects many signaling molecules and systems such as TGF-β, NF-κB, activator protein-1 (AP-1), the transcription factor specificity protein-1 (SP-1), and the renin–angiotensin system through AGE/RAGE signaling, which provokes chronic inflammation, glomerular and tubular hypertrophy, and consequently augments renal failure (Lee and Park, 2013; Tiwari et al., 2013). The activation of Nrf2 significantly protects against DN via its translocation into the nucleus, where it triggers the modulation of multiple DN biomarkers (collagen IV, laminin, TGF-β1, and fibronectin) (Adelusi et al., 2020). Numerous compounds that prevent DN through modulating the Nrf2 pathways, such as chlorogenic acid (CGA) and bardoxolone methyl, have already been tested in renal clinical trials (Thomas, 2012; Bao et al., 2018).

The typical pathological changes in DN are kidney fibrosis and renal tubular epithelial-to-mesenchymal transition (EMT), which eventually lead to ESRD. Several vital signals participate in fibrotic changes, including GBM thickening, mesangial matrix widening, glomerular sclerosis, renal tubular basement membrane thickening, and renal interstitial fibrosis (Hoshino et al., 2015). Although the pathogenesis of DN fibrosis is not clear, TGF-β is proved to be a major regulator of EMT and the possible cause of an excessive accumulation of the ECM via a variety of intracellular signaling molecules such as protein kinases and cytokines (Sutariya et al., 2016).

In fact, multiple molecules participate in the pathophysiologic process of DN, and the definitive mechanisms of PTMs on these molecules are intricate. Due to space limitations, we only review the effects of PTMs on NF-κB, Nrf2, and TGF-β during DN.

## The Effect of Ptms on Central Signals in DN

A series of pathways, including the NF-κB, Nrf2, and TGF-β signaling pathways, are activated by major pathophysiological processes in DN such as hyperglycemia, oxidative stress, advanced glycation end products (AGEs), and angiotensin II. Recent studies demonstrated that these pathways were modulated via the ubiquitin–proteasome system (UPS) and small ubiquitin-like modifier (SUMO), which were involved in the occurrence and progression of DN (Rotin and Staub, 2011; Gao et al., 2014).

### The Role of Ubiquitylation and SUMOylation in DN

#### A Glance at Ubiquitylation and SUMOylation

The UPS for endogenous protein degradation is characterized by its high efficiency and high selectivity for the degradation of specific proteins in eukaryotic cells. The UPS carries out two functions: the ubiquitylation of target proteins and the degradation of target proteins in the proteasome. The UPS involves ubiquitin, the ATP-dependent activating enzyme E1, the ubiquitin carrier protein E2, the ubiquitin-protein ligase enzyme E3, and the proteasome. The efficiency and complexity of the UPS determine its importance in the modification of cellular functions such as cell signaling, protein trafficking, DNA repair, chromatin modifications, cell cycle progression, and cell death, which are involved in DN during physiological and pathological conditions. Members of the UPS, such as cullin-1, cullin-3, and the 11S proteasome regulators PA28-β and PA28-γ, were upregulated in the intraglomerular capillaries composed of endothelial cells, basement membrane, and epithelial cells of mice with DN (Aghdam et al., 2013). Besides, it was reported that the inhibition of the systemic proteasome ameliorated renal pathologies (Gao et al., 2014). These pieces of evidence suggested that modulation of the UPS could prevent DN.

SUMO polypeptides are approximately 18% identical to ubiquitin at the amino acid sequence level, and their three-dimensional (3D) structural folds are highly similar to that of ubiquitin. Four hypotypes (SUMO-1,2,3,4) have been found in mammalian cells (Yang and Chiang, 2013). SUMOylation, a transient protein modification mediated by the SUMO peptide, modifies various eukaryotic proteins in organisms ranging from yeast to humans and can exert distinct biological functions by attaching the SUMO peptide to substrates in both its monomeric and polymeric forms (Geiss-Friedlander and Melchior, 2007). SUMOylation also leads to an enzymatic cascade resembling that of ubiquitylation (Rytinki et al., 2009). SUMO attachment is a reversible and highly transient modification partly due to the important fact that the same enzymes facilitating the initial attachment of SUMO molecules can catalyze their cleavage from the substrate.

An association between SUMOylation and the ubiquitin pathway is not surprising based on the close relationship between the two proteins. Since SUMO peptides can be attached to the same lysine residues as ubiquitin, the SUMOylation of a substrate protein may protect against degradation in the ubiquitin pathway, which consequently leads to competition for attachment at the same lysine residue on the substrate. In addition, protein sumoylation is a highly dynamic process in the cell, closely related to the different endings for the substrate and consequently different cellular activities. For example, IκBa is the first SUMO-modified protein implicated in NF-κB regulation to induce multiple critical roles in regulating both initial activation of NF-κB and the duration of this activity in response to extracellular signals, while SUMO-modified protein substrate in the NF-κB signaling pathway is NEMO when cells face DNA-damaging agents (Mabb and Miyamoto, 2007; Liu et al., 2013). In addition to the competitive nature of these two PTMs, the SUMO/ubiquitin interplay can also cooperate to produce a synergic outcome for the ubiquitin–proteasome pathway. This is because the process in which SUMO modification serves as a targeting signal for the ubiquitin–proteasome pathway occurs in a sequential manner. For example, Guzzo et al. (2012) reported SUMO-Ub chains synthesized by RNF4-targeted PML (promyelocytic leukemia protein) for PML proteasomal degradation in humans. Besides, the relationship between the SUMO and ubiquitin pathways is even more intricate considering that the enzymes of one pathway can be regulated by the other (Sriramachandran and Dohmen, 2014).

#### The Modulation of NF-κB With Ubiquitylation and SUMOylation

Nuclear factor kappa-B bound to v-rel avian reticuloendotheliosis viral oncogene homolog (REL) forms the NF-κB complex, which is inhibited by inhibitory κB (IκB) proteins. Ubiquitylation-mediated phosphorylation of serine residues on IκB leads to the detachment of IκB from NF-κB, which consequently allows the activation of the NF-κB complex. The activated NF-κB complex translocates into the nucleus and binds DNA at NF-κB-binding motifs to exert a pro-inflammatory effect during DN. Chen et al. (2012) found a significant simultaneous increase in the expression of NF-κB in the nucleus and ubiquitin in the cytoplasm of glomerular cells composed of glomerular filtration membrane and mesangial membrane in rats with DM compared to their expression in normal rats, suggesting that ubiquitin participates in NF-κB-related inflammation and consequently contributes to renal damage in rats with DM. Guo and Peng indicated that MG132, a specific, potent, and reversible proteasome inhibitor, blocked the degradation of ubiquitin-conjugated IκB and thereby inhibited NF-κB activation (Guo and Peng, 2013).

Moreover, the interaction between SUMO and IκB or NF-κB is responsible for the occurrence and development of inflammation contributing to renal injury (Siednienko and Gorczyca, 2003; Aghdam and Sheibani, 2013). The significance of SUMO in the regulation of NF-κB is also demonstrated. Mabb and Miyamoto (2007) detected that most of the signal transduction molecules in the NF-κB pathway, such as IκBα, p100, RelA, and NEMO, can be modified by SUMOylation. Lukic et al. (2013) indicated that SUMO-1 is directly conjugated to IκBα at residues K21 and K22, which prevents IκBα from signal-induced ubiquitylation and degradation and thus limits NF-κB activation. Wang et al. (2009) observed that the translocation of NF-κB (p65) and IκBα into the nucleus and the expression of SUMO-4 and IκBα were significantly higher in the nucleus of the kidneys of diabetic GK rats, which indicated the role of SUMO-4 in negatively regulating NF-κB signaling in glomerular endothelial cells. The SUMOylation of p100 is the primary step in the activation of NF-κB; thus, blocking the SUMOylation of p100 can inhibit the ultimate activation of this pathway (Vatsyayan et al., 2008). Moreover, Liu et al. (2012) provided the first experimental evidence that PIAS3, a member of the protein inhibitor of activated STAT (PIAS) protein family with E3 SUMO ligase activity, SUMOylated the RelA subunit of NF-κB, consequently inducing NF-κB repression. This interaction is induced by NF-κB activation, forming a negative regulatory loop (Liu et al., 2012). It was also reported that the SUMO-1 modification of NEMO (NF-κB essential modulator) is related to the upregulation of NF-κB activation in response to genotoxic stress, including DNA lesions, unusual secondary DNA structures, limiting nucleotide levels, and interference from transcription (Kfoury et al., 2011). These findings emphasize the importance of ubiquitylation and SUMOylation in the modulation of NF-κB in DN.

#### The Modulation of Nrf2 With Ubiquitylation and SUMOylation

Nrf2 plays a vital role in DN by increasing the gene expression of antioxidant enzymes such as superoxide dismutase (SOD), glutathione peroxidase (GPx), catalase (CAT), and nicotinamide adenine dinucleotide phosphate [NAD(P)H] in the nucleus to prevent oxidative stress (Rytinki et al., 2009).

Kelch-like ECH-associated protein 1 (Keap1), an inhibitor of Nrf2, constantly recruits Nrf2 to form dimers by binding to two different motifs on Nrf2, the ETGE, and DLG motifs. Keap1 is a substrate adaptor for cullin-3 to form the E3 ubiquitin ligase complex, inducing the ubiquitin-related degradation of Nrf2 under normal conditions (Tong et al., 2007; Cui et al., 2013a; Niture et al., 2014). The overproduction of ROS during DN weakens the interaction between Keap1 and the DLG motif of Nrf2, which impairs the Keap1-mediated ubiquitylation of Nrf2 and thereby activates Nrf2 (Jiang et al., 2016). Luo et al. (2011) discovered a series of improvements in rats with DN after treatment with MG132, including the 24-h urinary protein excretion rate (UPER) and renal pathological changes, which was consistent with the results of numerous studies (Kong et al., 2017). Levels of the renal 26S proteasome, p47phox, and nitrotyrosine (NT) were reduced in rats with DN by MG132 treatment. These changes were closely related to the activation of Nrf2 via MG132-inhibited ubiquitylation and the consequent upregulation of renal SOD1, CAT, and GPx in DN rats (Luo et al., 2011). However, the effects of PA28 (a proteasome activator) on DN are still controversial. Studies have shown that the initially increased level of PA28 in the glomerulus is also synchronized with oxidative stress in DN, which might play a protective role against oxidative damage (Koya et al., 2003), while the chronic activation of PA28 exacerbates the pathogenesis of DN (Singh et al., 2011).

The discovery of RNF4 and RNF11, the only two known human polySUMO-specific E3 ubiquitin ligases [also called SUMO-targeted ubiquitin ligases (STUbLs)], provided the first direct link between SUMO modification and the ubiquitin/proteasome system (Kawai et al., 2011; Singh et al., 2011; Sriramachandran and Dohmen, 2014). These two STUbLs modulate Nrf2 in a Keap1-independent manner while exerting different functions. RNF4 mediated the polyubiquitylation of polySUMOylated Nrf2, leading to its subsequent degradation in promyelocytic leukemia nuclear bodies (PML NBs) (Malloy et al., 2013). However, RNF11 positively regulates nuclear Nrf2 levels during oxidative stress through the RNF11-mediated s-linked ubiquitylation of Nrf2, which results in the stabilization of Nrf2 within PML NBs (McIntosh et al., 2018). In addition, Chowdhry et al. (2013) reported another Keap1-independent function of Nrf2; the Neh6 domain of Nrf2 contains two degrons that can mediate Nrf2 degradation via the E3 ubiquitin ligase β-TrCP. These Keap1-independent mechanisms of Nrf2 regulation provide significant protection against DN and require further research for effective therapy.

#### The Modulation of TGF-β With Ubiquitylation and SUMOylation

Transforming growth factor-β is a critical factor in DN fibrosis through inducing glomerular and tubular cell hypertrophy and ECM accumulation, promoting glomerular sclerosis and renal interstitial fibrosis, and interacting with high glucose, angiotensin II (Ang II), and other profibrotic factors (Ziyadeh, 2004; Ko et al., 2013).

**FIGURE 1:**
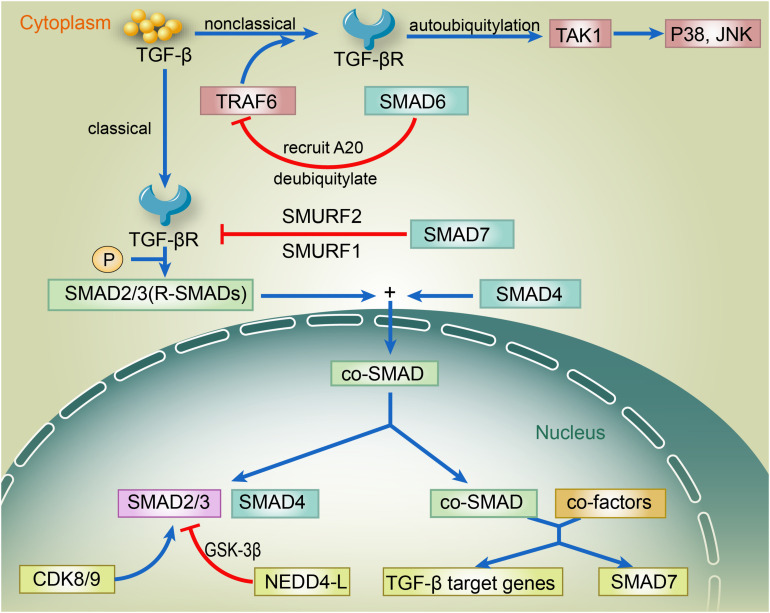
The regulation of TGF-β via canonical and non-canonical pathways. In the canonical pathway, binding of TGF-β ligand to TGF-βR induces the phosphorylation of SMAD2/3. Phosphorylated SMAD2/3 forms a complex with SMAD4 and then translocates to the nucleus. Once it enters the nucleus, the SMAD2/3–SMAD4 complex binds to other transcriptional co-factors, which initiates the transcription of TGF-β target genes and the expression of SMAD7. SMAD7 recruits SMURF1 and SMURF2 to the TGF-βR and induces its proteasome-dependent degradation, which eventually weakens the TGF-β signaling. Inside the nucleus, the linker region of SMAD2/3 is phosphorylated by CDK8 and CDK9, enhancing the TGF-β signaling by promoting the binding of SMAD2/3 to other transcriptional coactivators. The GSK-3β-phosphorylated SMAD2/3 can be recognized by NEDD4-L and then undergo the ubiquitin-mediated degradation, thereby attenuating the TGF-β signaling. In the non-canonical pathway, binding of the TGF-β ligand to its receptor promotes the binding of TRAF6 to the TGF-βRs. TRAF6-induced autoubiquitylation of TGF-βRs activates TAK1, which further triggers the downstream p38 and JNK pathways. TGF-β, transforming growth factor β; TGF-βR, TGF-β receptor; SMAD; SMURF, SMAD ubiquitylation regulatory factor; CDK, cyclin-dependent kinase; GSK-3β, glycogen synthase kinase-3β; NEDD4-L, neural precursor cell expressed developmentally down-regulated 4-like; TRAF6, TNF receptor-associated factor; TAK1, transforming-growth-factor-β-activated kinase; JNK, c-JUN N-terminal kinase.

The regulation of TGF-β by ubiquitylation and SUMOylation is achieved by the modification of enzymes related to TGF-β within its canonical and non-canonical pathways (Iyengar, 2017). The canonical pathway involves SMAD2, SMAD3, SMAD4, SMAD7, and SMAD regulatory factor 2 (SMURF2), while the non-canonical pathway mainly involves TNF receptor-associated factor 6 (TRAF6), Smad6, A20, transforming growth factor-β (TGF-β)-activated kinase 1 (TAK1), p38, and c-Jun amino-terminal kinase (JNK). In the canonical pathway, binding of TGF-β ligand to TGF-βR induces the phosphorylation of SMAD2/3. Phosphorylated SMAD2/3 forms a complex with SMAD4 and then translocates to the nucleus. Once it enters the nucleus, the SMAD2/3–SMAD4 complex binds to other transcriptional co-factors, which initiates the transcription of TGF-β target genes and the expression of SMAD7. SMAD7 recruits SMURF1 and SMURF2 to the TGF-βR and induces its proteasome-dependent degradation, which eventually weakens the TGF-β signaling. Inside the nucleus, the linker region of SMAD2/3 is phosphorylated by CDK8 and CDK9, enhancing the TGF-β signaling by promoting the binding of SMAD2/3 to other transcriptional coactivators. The GSK-3β-phosphorylated SMAD2/3 can be recognized by neural precursor cell expressed developmentally downregulated 4-like (NEDD4-L) and then underwent the ubiquitin-mediated degradation, which thereby attenuates the TGF-β signaling. In the non-canonical pathway, binding of the TGF-β ligand to its receptor promotes the binding of TRAF6 to the TGF-βRs. TRAF6-induced autoubiquitylation of TGF-βRs activates TAK1, which further triggers the downstream p38 and JNK pathways (Figure 1). Ubiquitin-specific protease 15 (USP15) regulates TGF-β differently depending on its targets. USP15 is responsible for the positive regulation of TGF-β signaling to deubiquitylate TGF-β receptor and rescue it from SMURF2-mediated degradation (Eichhorn et al., 2012). USP15 also binds to SMAD7, forming a complex with SMURF2 that negatively regulates TGF-β. Also, USP15 is shown to deubiquitylate SMURF2 at K345, K412, K615, K620, K687, and K734, which is critical for SMURF2 activity. The deubiquitylation of SMURF2 consequently attenuated the inhibitory effect of SMURF2 on TGF-β (Iyengar et al., 2015). Furthermore, the SUMOylation of SMURF2 upregulated the TGF-β signal (Hay, 2005). TRAF4 and SMURF2 are antagonists, which illustrates the positive correlation between TRAF4 and TGF-β (Zhang et al., 2013). However, previous TRAF4-deficient cell studies reported that the depletion of TRAF4 enhanced cell–cell adherence, decreased cell migratory capacity, and improved EMT changes (Carpentier et al., 2008; Kalkan et al., 2009; Wang et al., 2013). SMAD6 recruited A20 to deubiquitylate TRAF6, consequently inducing the inactivation of TRAF6, which negatively regulated the non-canonical TGF-β pathway (Sorrentino et al., 2008; Jung et al., 2013). RNF11 is a positive regulator of TGF-β via inducing the ubiquitylation and degradation of SMAD7, SnoN, and c-Ski (transcriptional corepressors) to enhance TGF-β signaling (Koinuma et al., 2003; Nagano et al., 2007).

### The Effect of Other PTMs on NF-κB, Nrf2, and TGF-β

#### Phosphorylation

High glucose levels induce the phosphorylation and degradation of inhibitory IκB proteins, followed by the liberation and phosphorylation of NF-κB and the consequent NF-κB nuclear translocation and DNA binding. Treatment with oridonin (a component isolated from *Rabdosia rubescens*) inhibited IκBα phosphorylation and p65 phosphorylation and was involved in suppressing NF-κB, which is responsible for the increase in inflammatory mediators and cytokine production in DN (Li et al., 2018).

The phosphorylation of Nrf2 at its transcription activation (TA) domains Neh4 (Nrf2-ECH homology 4) and Neh5 (Nrf2-ECH homology 5) by CK2 triggered Nrf2 activation, which was necessary for the nuclear localization and transcriptional activation of Nrf2. The RNA-like endoplasmic reticulum kinase (PERK)-dependent phosphorylation of Nrf2 induced the dissociation of cytoplasmic Nrf2/Keap1 complexes and subsequent Nrf2 nuclear translocation (Cullinan et al., 2003). Mitogen-activated protein kinases (MAPKs) phosphorylated Nrf2 at multiple serine/threonine residues, which induced a limited modulation in Nrf2 activity and did not alter Nrf2 protein stability (primarily controlled by Keap1) (Sun et al., 2009). However, Rada et al. (2011) first reported the degradation of Nrf2 after it was targeted by glycogen synthase kinase 3 (GSK-3) in an E3 ligase SCF/β-TrCP-dependent and Keap1-independent manner.

The phosphorylation of multiple key molecules within the TGF-β pathway plays an essential role in TGF-β modulation. Phosphorylated pSmad2 is a molecular marker of TGF-β/activin activity (Hoj Thomsen et al., 2017). Besides, the dose-dependent inhibition of TGF-β RII and Smad2 phosphorylation in rat kidneys by niclosamide demonstrated the protective effect of niclosamide against podocyte injury, which might be due to the suppression of TGF-β-involved fibrosis (Zhang et al., 2017).

Molecules outside the TGF-β pathway modulate TGF-β as well. For example, lysophosphatidic acid (LPA) receptor 1 signaling increased TGF-β expression via GSK-3β phosphorylation and sterol regulatory element-binding protein 1 (SREBP1) activation, which contributed to the development of DN (Li et al., 2017).

#### Glycosylation

The NF-κB subunit c-Rel was modified and activated via the addition of *O*-linked β-*N*-acetylglucosamine (a process known as *O*-GlcNAcylation), an abundant PTM in hyperglycemic conditions in DN (Ramakrishnan et al., 2013). *O*-GlcNAcylation of target proteins requires UDP-GlcNAc, a nucleotide sugar derived from glucose and other metabolites via the hexosamine biosynthetic pathway, which directly links nutrient status to *O*-GlcNAc signaling [21391816 25825515]. Chen et al. (2012) link nutrient sensing to redox stress signaling (Nrf2 signaling) through glycosylation of Keap1, with the exciting discovery that the *O*-GlcNAcylation of Keap1 at serine 104 to promote its productive interaction with CUL3 is required for the efficient ubiquitylation and degradation of Nrf2 [28663241]. Furthermore, they noted that Keap1 S104 *O*-GlcNAcylation can lead to efficient downstream ubiquitination of Nrf2. They further tested the correlation between Nrf2, *O*-GlcNAc, and glucose level and found that glucose deprivation reduces Keap1 glycosylation and decreases the productive Keap1–CUL3 interaction, leading to Nrf2 pathway activation, suggesting that glucose contributes to keap1 glycosylation.

Wang et al. (2017) found that core fucosylation catalyzed by fucosyltransferase 8 (FUT8) regulated the pericyte–myofibroblast transition during renal interstitial fibrosis (RIF) by activating both the TGF-β/SMAD and platelet-derived growth factor (PDGF)/extracellular signal-regulated kinase (ERK) pathways. Chan et al. (2018) reported that the abnormal functioning of underglycosylated proteoglycans, including decorin and biglycan, was involved in TGF-β activation and a disorganized collagen network in Gerodermia osteodysplastica.

#### Acetylation

The covalent modification and acetylation of Nrf2 induced by CREB-binding protein increased the capacity of Nrf2 to bind to its cognate response element in a target gene promoter and the Nrf2-dependent transcription from target gene promoters after its release from Keap1 (Kawai et al., 2011). Ishinaga et al. (2007) reported that TGF-β triggered the acetylation of p65 at lysine 221 via a Smad3/4-PKA-p300-dependent signaling pathway, which was closely related to NF-κB activation and the consequent NF-κB-dependent transcription of TNF-α and IL-1β and interstitial polymorphonuclear neutrophil infiltration *in vitro* and *in vivo*.

#### Methylation

A series of studies indicated that the reversible methylations of lysine or arginine residues in NF-κB at six methylated K sites (K37, 218, 221, 310, 314, and 315) are modulated by histone-modifying enzymes including lysine and arginine methyltransferases and demethylases. These methylations are also necessary to activate many downstream genes such as TNF-α, IL-1, and Toll-like receptor ligands (Ea and Baltimore, 2009; Wei et al., 2013; Lu and Stark, 2015).

Although the role of Nrf2 and TGFβ methylation in transcriptional modifications has been reported, the posttranslational methylation of these two molecules remains to be thoroughly studied.

## The Roles of Central Signaling Pathways in the Common Abnormal Cellular Activities in DN

### Inflammation and NF-κB in DN

#### Inflammation in DN

Recently, increasing numbers of studies have clarified the central role of inflammation in the pathogenesis of DN *in vivo* and *in vitro* through various pro-inflammatory factors, including NF-κB, monocyte chemoattractant protein-1 (MCP-1), TNF-α, and interleukin-1β (IL-1β) (Elmarakby and Sullivan, 2012; Sun and Kanwar, 2015; Sun et al., 2016; Yang et al., 2016). In particular, innate immune responses facilitate the inflammatory process of DN and induce macrophage and T cell infiltration to the glomeruli and interstitium, which promotes the local release of adhesion molecules and chemokines (Tuttle, 2005). It was shown that macrophages are the major contributor among infiltrating leukocytes to the impairment of renal function in patients with DN, especially CD68+ macrophages, which make up 90% of the total kidney leucocyte infiltrate (Chow F. et al., 2004; Chow F. Y. et al., 2004; Nguyen et al., 2006). Infiltrating macrophages interact with resident renal cells and induce chronic, low-grade inflammation, promoting the synthesis of pro-inflammatory cytokines, namely, IL-1β, IL-6, IL-18, and TNF-α, and MCP-1 (Sugimoto et al., 1999). Toll-like receptor 4 (TLR4) is the crucial factor contributing to the aggravation of renal dysfunction via regulating central pathways in DN (Akira et al., 2001). The activation of TLR4 participates in the pathogenesis of DN by transmitting a signal via an adaptor molecule, MyD88, leading to the translocation of NF-κB and p38-MAPK as well as the subsequent upregulation of pro-inflammatory cytokines and chemokines (Ma et al., 2014).

#### The Role of NF-κB in DN

The activation of NF-κB is induced by a wide variety of stimuli, such as cytokines, oxygen radicals, inhaled particles, ultraviolet irradiation, and bacterial or viral products. NF-κB expression is induced by hyperglycemia (HG), and NF-κB is activated in peripheral blood mononuclear cells and in kidney biopsy specimens. Proteinuria serves as an important activator of NF-κB and an important pro-inflammatory stimulus for tubular epithelial cells in DN. Increasing numbers of studies have reported that NF-κB plays a significant role in the inflammatory response in the kidneys of patients with progressive DN (Pichler et al., 2017).

Nuclear factor kappa-B activation participates in expressing a wide variety of HG-induced inflammatory genes, including genes encoding adhesion molecules, chemokines, and inflammatory cytokines. IL-1β, IL-6, and TNF-α expression are partially dependent on NF-κB, which leads to DN. In addition to the factors mentioned above, NF-κB binds to the promoter regions of several other genes that are involved in the pathogenesis of DN, such as those encoding TGF-β1, aldo-keto reductase family 1, member B1 (AKR1B1), CC chemokine ligand 2, which is also known as MCP-1 (monocyte chemoattractant protein-1) (CCL2), and intercellular adhesion molecule 1 (ICAM-1) (Park et al., 2000; Goldberg et al., 2006; Nam et al., 2008; Yang et al., 2008). NF-κB is also integrated into various biological pathways functionally involved in the pathogenesis of DN, such as protein kinase C (PKCβ) signaling, the RAS pathway, AGE accumulation, and oxidative stress. The suppression of NF-κB activation by various agents such as thiazolidinediones, 1,25-dihydroxyvitamin D3, cilostazol, oridonin, and curcumin could lead to the improvement of DN, suggesting the significance of NF-κB as a therapeutic target in DN (Kumar et al., 2001; Lee et al., 2004, 2010; Pillarisetti and Saxena, 2004; Ohga et al., 2007; Zhang et al., 2007; Liang et al., 2010; Soetikno et al., 2011; Li et al., 2018).

#### Other Pathways Related to Inflammation in DN

Besides the pathways related to NF-κB, other pathways also contribute to DN, such as the MAPK pathway. HG-induced MAPK activation plays an important role in DN development by modulating various transcription factors via phosphorylation and inducing the expression of genes encoding pro-inflammatory molecules, including TNF-α, IL-1β, and IL-6 (Ma et al., 2009; Tesch, 2017). Thus, targeting the p38-MAPK signaling pathway is an attractive strategy for developing anti-inflammatory drugs to treat DN.

### Oxidative Stress and Nrf2 in DN

#### Oxidative Stress in DN

Oxidative stress plays a significant role in the pathological process of DN, which is related to changes in the equilibrium of the redox state caused by a persistent hyperglycemic state and the increase in AGEs.

The interaction between AGEs and RAGEs induces the proliferation, apoptosis, autophagy, or migration of cells depending on the target cell, which incites the intracellular production of ROS. Interestingly, it was found that oxidative stress facilitates the polyol pathways to promote the generation of AGEs such as *N*-carboxymethyl-lysine and pentosidine, which consequently form an interaction loop between ROS and AGEs (Mangalmurti et al., 2010; Delbin et al., 2012; Lo et al., 2015; Barandalla et al., 2017). These events affect the renin–angiotensin system and regulate TGF-β, NF-κB, AP-1, and SP-1, which provokes abundant pro-inflammatory and profibrotic responses. This suggests that the treatment of DN should be focused not only on the early control of glycemia but also on the limitation of factors related to oxidative stress and the formation of AGEs (Shao et al., 2013).

#### The Contribution of Nrf2 in DN

Nrf2 plays a significant role in DN, which is closely related to the discovery that the conspicuous impairment of Nrf2 in DM contributes to the severity of oxidative stress, inflammation, fibrosis, and the progression of tissue damage in the kidney (Zheng et al., 2011). Decreased antioxidant activity already existed at an early stage of DN, and that antioxidant capacity weakened in parallel with the severity of kidney disease in diabetic rats (Shao et al., 2013). Hence, the therapeutic activation of Nrf2 already shows the power to prevent or slow the progression of DN (Zheng et al., 2011).

Nrf2 modulators, including maxacalcitol (an active vitamin D analog), sulforaphane, chlorogenic acid (CGA), epigallocatechin gallate, and sitagliptin, exert a protective effect on DN by suppressing oxidative stress (Cui et al., 2012; Nakai et al., 2014; Civantos et al., 2017; Sun et al., 2017; Bao et al., 2018). The significance of H_2_S in DN is related to numerous pathways and not only to Nrf2. H_2_S reduces high glucose-induced oxidative stress by activating the Nrf2 antioxidant pathway, exerts anti-inflammatory effects by inhibiting NF-κB signaling, reduces high glucose-induced mesangial cell proliferation by blocking MAPK signaling pathways, and inhibits the renin–angiotensin system in diabetic kidneys (Zhou et al., 2014).

Besides oxidative stress, other biological oxygen anomalies such as hypoxia have been implicated in DN (Miyata et al., 2013). Diminished Nrf2 mRNA and protein expression was found in mice after 8 weeks of intermittent hypoxia (Wu et al., 2015). HIF activates a broad range of reactions against hypoxia. Gu et al. (2013) first demonstrated the protective effect of the HIF-1A Pro582Ser polymorphism in DN, which possibly confers resistance to the repressive effect of glucose on HIF-1α.

Although the protective effect of Nrf2 has been reported, scholars have observed that the constitutive overactivation of Nrf2 is associated with cancer development and progression and chemotherapy resistance in human bronchial epithelial cells. This suggests that tight control of Nrf2 signaling may benefit DN (Yang et al., 2015).

### Fibrosis and TGF-β in DN

#### Fibrosis in DN

Diabetic nephropathy is characterized by a variety of renal morphological changes, including the following: GBM thickening, mesangial matrix widening, glomerular sclerosis, podocyte loss, renal tubular basement membrane thickening, tubular atrophy, and increased apoptosis, renal inflammatory infiltration, renal interstitial fibrosis, sparse periosteal capillaries, and the hyaline degeneration of the walls of afferent and especially efferent arterioles (Hoshino et al., 2015). Although the pathogenesis of DN is not clear, renal fibrosis resulting from the excessive accumulation of the ECM is often regarded as a major contributor to end-stage renal failure in DN.

#### The Contribution of TGF-β in DN

In DN, a series of factors regulate TGF-β expression in renal cells, including hyperglycemia, angiotensin II, AGEs, complement activation (C5b-9), and oxidative stress. TGF-β plays a significant role in DN development because it can regulate EMT and may promote fibrosis via numerous intracellular signals such as protein kinase and cytokines (Sutariya et al., 2016). For example, TGF-β can send a signal through ALK1 or ALK5, leading to the phosphorylation of SMAD1/5 or MAD2/3, respectively (Massague, 2000; Lebrin et al., 2005; Scherner et al., 2007; Velasco et al., 2008). Oujo et al. (2014) found an increase in SMAD2/3 phosphorylation in the tubular interstitium but not in tubular cells, suggesting that SMAD2/3 phosphorylation is related to changes in interstitial myofibroblast cells rather than in epithelial cell function. Also, a meta-analysis of randomized controlled trials revealed that serum TGF-β1 could act as a biomarker for DN (Mou et al., 2016).

Numerous studies have indicated that TGF-β target regulators such as resveratrol (RSV), *Salvia miltiorrhiza*, *Taxus chinensis*, and calcitriol improve fibrosis in DN via complex signaling pathways (Yu et al., 2015; Qiao et al., 2017; Weng et al., 2018; Xiang et al., 2018).

## Potential Directions of DN Treatment

Current clinical measures to prevent or delay DN include strict metabolic control, strict blood pressure control using angiotensin-converting enzyme inhibitors and angiotensin receptor blockers, tight control of serum lipids using statins as indicated, a low protein diet, the avoidance of smoking and other nephrotoxic influences, the prevention of abnormalities in calcium/phosphorus metabolism, and the prevention of renal anemia by the early use of erythropoietin (Tzamaloukas and Murata, 2005). However, more substances or medicines targeting the mechanisms mentioned above, such as MG132, RSV, atrasentan, and minocycline, show enormous prospects for DN treatment and even diabetes (Cui et al., 2013b; Shahzad et al., 2016; Pena et al., 2017). We suspect that the superposition of these substances may lead to greater therapeutic effects, which requires verification.

Additionally, the development of more mechanisms to diagnose DN is underway because the early diagnosis and screening of diabetes and DN have an important influence on prognosis (Garg et al., 2015). Garg et al. (2015) found that tubular damage may play a major role in developing nephropathy in prediabetes. Early markers such as urine cystatin C are increased early in diabetes and in preDN. In addition, urinary peptides are fragments of proteins that might serve as better markers of kidney injury in diabetes because their corresponding full-length proteins are only found in urine once advanced renal damage has occurred due to their larger size. These smaller urinary peptides pass more easily through the glomerular filter and could serve as early indicators of renal damage (Fu et al., 2016). Xanthosine and N1-methylguanosine were also reported to predict the development of nephropathy in T2DM patients (Chen et al., 2018). Given that several studies have reported the specific mechanism of polyubiquitination of Nrf2 and NF-κB, the relative molecules in these mechanisms performed great diagnostic and therapeutic value for DN (Huang et al., 2017; Gong et al., 2018).

## Conclusion

**FIGURE 2:**
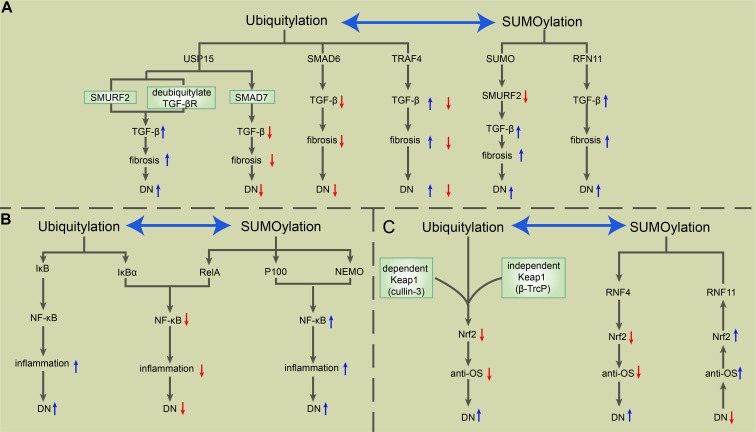
The roles of ubiquitylation and SUMOylation in the modulation of TGF-β, NF-κB, and Nrf2 in DN. **(A)** USP15 deubiquitylates TGF-βR and rescues it from SMURF2-mediated degradation, which induces the upregulation of TGF-β signaling and consequent exacerbation of fibrosis and DN. USP15 also binds to SMAD7, forming a complex with SMURF2 that negatively regulates TGF-β. SMAD6 recruits A20 to deubiquitylate TRAF6, consequently inducing the inactivation of TRAF6, which negatively regulates the non-canonical TGF-β pathway. The SUMOylation of SMURF2 upregulates the TGF-β signal. RNF11 is a positive regulator of TGF-β, positively correlated with fibrosis and the severity of DN. **(B)** Ubiquitylation-mediated phosphorylation of IκB allows the activation of the NF-κB complex, which exerts a pro-inflammatory effect during DN. SUMO-1 is directly conjugated to IκBα at residues K21 and K22, which prevents IκBα from SUMOylation-mediated IκBα degradation. Besides, RelA induces NF-κB repression, while SUMOylation-mediated p100 and NEMO promote NF-κB activation. **(C)** Keap1-mediated ubiquitylation of Nrf2 via cullin 3 and the Keap1-independent function of Nrf2 via β-TrCP degrade Nrf2 and weaken the antioxidative stress ability, which in turn exacerbates DN. RNF4 mediates the polyubiquitylation of polySUMOylated Nrf2, leading to its subsequent degradation in PML NBs. However, RNF11 positively regulates nuclear Nrf2 levels during oxidative stress through the RNF11-mediated s-linked ubiquitylation of Nrf2, which results in the stabilization of Nrf2 within PML NBs. NF-κB, nuclear factor-kappa B; TGF-β, transforming growth factor β; Nrf2, NF-E2-related factor 2; DN, diabetic nephropathy; USP15, ubiquitin-specific protease 15; TGF-βR, TGF-β receptor; SMURF, SMAD ubiquitylation regulatory factor; TRAF6, TNF receptor-associated factor 6; RNF, really interesting new gene (RING) finger protein 11; NEMO, nuclear factor (NF)-κB essential modulator; PML NB, promyelocytic leukemia nuclear bodies.

**FIGURE 3:**
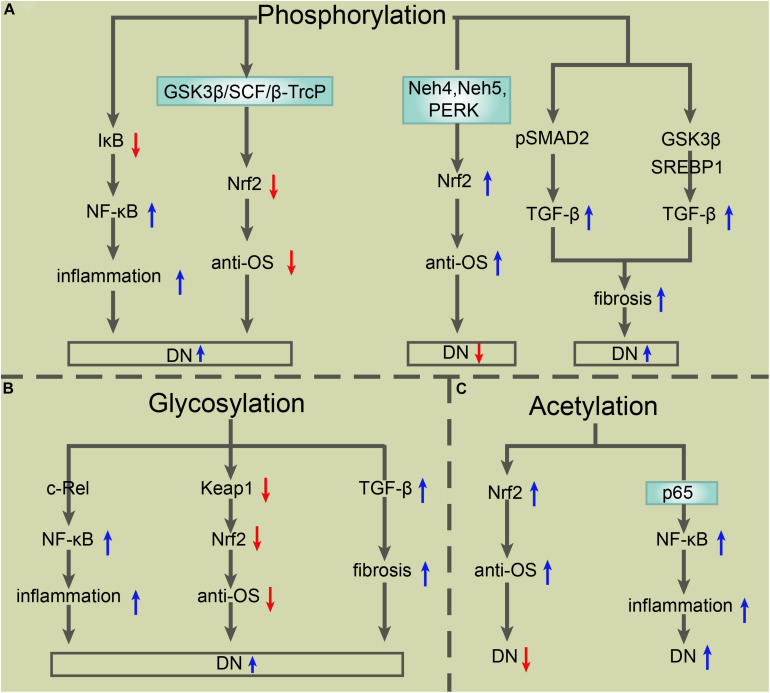
The roles of other PTMs in the modulation of TGF-β, NF-κB, and Nrf2 in DN. **(A)** Phosphorylation of IκB triggers its degradation, which results in the activation of NF-κB and consequent exacerbation of inflammation and DN. Phosphorylation-mediated Nrf2 degradation via GSK-3β in an E3 ligase SCF/β-TrCP-dependent manner exacerbates DN as well, while phosphorylation of Nrf2 via Neh4, Neh5, or PERK induces Nrf2 activation and consequent improvement of DN. Phosphorylated pSMAD2 or phosphorylation-mediated GSK3β/SREBP1 activation upregulates TGF-β expression, which enhances fibrosis and then exacerbates DN. **(B)** *O*-GlcNAcylation of the subunit c-Rel results in NF-κB activation, which exacerbates inflammation and DN. *O*-GlcNAcylation of Keap1 at serine 104 induces the degradation of Nrf2 and consequent exacerbation of DN. Core fucosylation-mediated upregulation of TGF-β/SMAD promotes fibrosis and the progress of DN. **(C)** Acetylation of Nrf2 enhances its antioxidative stress capacity, which exerts protection for DN. Acetylation of p65 at lysine 221 is closely related to NF-κB activation, which aggravates the inflammatory response and DN. PTMs, posttranslational modifications; TGF-β, transforming growth factor β; NF-κB, nuclear factor-kappa B; Nrf2, NF-E2-related factor 2; DN, diabetic nephropathy; Neh4, Nrf2 ECH homology 4; PERK, protein kinase RNA-like endoplasmic reticulum kinase; GSK-3β, glycogen synthase kinase-3β; SREBP, sterol regulatory element-binding proteins; *O*-GlcNAc, *O*-linked β-*N*-acetylglucosamine.

In recent decades, PTMs have become a popular research topic. The particular PTMs on the core molecules of NF-κB, Nrf2, and TGF-β in DN provide abundant research targets that contribute to DN treatment improvement. Ubiquitylation and SUMOylation improve or exacerbate DN depending on their different target signals. Until now, apart from the SUMOylation of TGF-β leading to DN exacerbation, other ubiquitylation and SUMOylation modifications of NF-κB, Nrf2, and TGF-β promoted two outcomes of DN (see Figure 2). Common PTMs also include phosphorylation, glycosylation, and acetylation. The glycosylation of NF-κB, Nrf2, and TGF-β is blamed for the worsened DN outcome, while the phosphorylation and acetylation of these key molecules have a different effect on DN patients (see Figure 3).

In summary, the current data indicate a complex network of signaling mechanisms involved in DN mediation. However, many unsolved issues regarding the pathophysiological mechanisms of DN remain. Undoubtedly, more work is required to understand the definitive mechanisms of DN in renal cell biology under systemic hyperglycemic conditions to ensure that DN can be treated with more valuable therapies.

## Author Contributions

HT and ZJ designed and managed the project. YCa, ZY, ZW, and SJ collected the data, wrote the manuscript, and drew the figures. ZW, BD, and YY did the data processing and quality control. All authors discussed, critically revised, and approved the final version of the manuscript.

## Conflict of Interest

The authors declare that the research was conducted in the absence of any commercial or financial relationships that could be construed as a potential conflict of interest.

## Footnotes

1 http://www.idf.org/diabetesatlas
